# Arc Discharge Synthesis and Photoluminescence of 3D Feather-like AlN Nanostructures

**DOI:** 10.1007/s11671-010-9745-4

**Published:** 2010-08-26

**Authors:** SL Yang, RS Gao, PL Niu, ZY Zou, RH Yu

**Affiliations:** 1 Department of Materials Science and Engineering, Tsinghua University, Beijing, 100084, People's Republic of China; 2 Astronaut Center of China, Beijing, 100094, People's Republic of China; 3 School of Materials Science and Engineering, Beihang University, Beijing, 100191, People's Republic of China

**Keywords:** Arc discharge, Feather-like AlN nanostructure, Alternate growth model, Photoluminescence

## Abstract

A complex three-dimensional (3D) feather-like AlN nanostructure was synthesized by a direct reaction of high-purity Al granules with nitrogen using an arc discharge method. By adjusting the discharge time, a coral-like nanostructure, which evolved from the feather-like nanostructure, has also been observed. The novel 3D feather-like AlN nanostructure has a hierarchical dendritic structure, which means that the angle between the trunk stem and its branch is always about 30° in any part of the structure. The fine branches on the surface of the feather-like nanostructure have shown a uniform fish scale shape, which are about 100 nm long, 10 nm thick and several tens of nanometers in width. An alternate growth model has been proposed to explain the novel nanostructure. The spectrum of the feather-like products shows a strong blue emission band centered at 438 nm (2.84 eV), which indicates their potential application as blue light-emitting diodes.

## Introduction

In recent years, the complex three-dimensional (3D) semiconductor nanostructures have become a research focus, which are considered as basic units of future nano-devices and ideal specimens for the fundamental physical research in nanoscale. Moreover, understanding the formation mechanism of these complex 3D nanostructures should lead to advances in nano-fabrication. AlN with a wide direct band gap (6.2 eV) is a quite important III–V semiconductor, which has some excellent physical and chemical properties, such as high heat conductivity, high hardness, high chemical stability and a similar thermal expansion with Si. In the past decade, AlN nanostructures have aroused much interest for promising application in field-emission (FE) and visible light emission. AlN nanowires and array [1-4], nanorod array [5-7], nanotips and array [8-11], nanobelts [12,13] and comb-like structures [14], which are classified as low-dimension nanostructures, have been synthesized by different methods. Recently, some complex 3D AlN nanostructures, called urchin-like [15], sixfold symmetry [16,17], pine-shaped [18] and flowerlike [9] nanostructures, have been reported. The formation of the complex AlN nanostructures was generally considered as a vapor–solid (VS) process. To the best of our knowledge, there has been no report on the synthesis of high-quality complex 3D feather-like AlN nanostructures.

In this paper, a 3D feather-like AlN nanostructure has been prepared by a direct arc discharge method [5,9,15] without any catalyst or template, and an alternating growth mechanism is introduced to understand the novel nanostructure. By extending the discharge time, a coral-like nanostructure, which evolved from the feather-like nanostructure, has also been prepared. The spectrum of photoluminescence reveals that the feather-like AlN nanostructure has an excellent blue emission band centered at 438 nm (2.84 eV), indicating potential applications in optoelectronic nano-devices.

## Experimental Approach

High-purity Al granules (99.99%) were arc melted to a pure Al ingot in a pure argon atmosphere. Before arc discharge, the chamber was evacuated to 5 × 10^-3^ Pa and flushed with pure Ar two times to remove oxygen. Then, a mixture of pure nitrogen and Ar was flushed in as reaction gas with a pressure of 10^5^ Pa. The ratio of partial pressure of N_2_ used in experiments was 50%. The tip of a tungsten cathode was kept about 10 mm above the Al ingot during the arc discharge. The discharge current and time were considered as two key parameters, which were adjusted in the experiments in order to investigate the growth of the products. When the discharge began, a pit would appear on the ingot immediately. After arc discharge, some gray products in the pit were collected for later characterization. The phase composition and chemical composition of the samples have been detected by X-ray diffraction (XRD; D8-ADVANCE) and energy-dispersive X-ray spectroscopy (EDS), respectively. The morphology and crystalline structure of the products were characterized by field-emission scanning electron microscopy (FE-SEM; Gemini LEO-1530) and transmission electron microscopy (TEM; JEOL JSM 2011). The photoluminescence spectrums of the samples were measured at room temperature using a spectrophotometer (PL, LS55, a Xe lamp with 325-nm wavelength as the excitation light source).

## Results and Discussion

**Figure 1:**
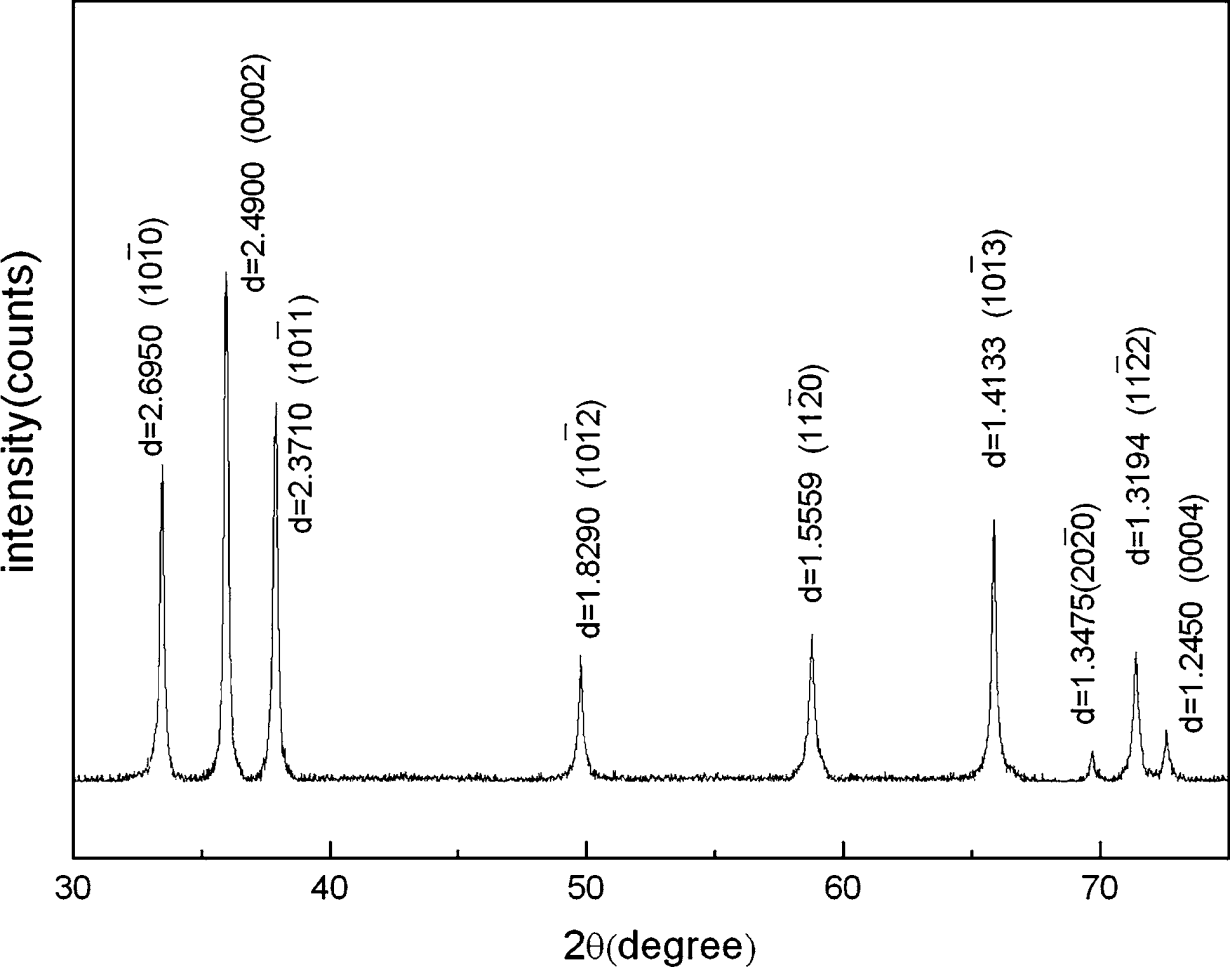
**XRD pattern of the products with all peaks indexed to wurtzite-structure AlN (h-AlN)**.

The arc discharge experiments have been carried out with a series of current, from 100 to 200 A. Amazing products had been prepared at 180 A, and the discharge time was set at 30 and 60 s. The as-grown products prepared by different discharge time have almost the same XRD pattern. Figure 1 shows a typical XRD pattern of the products. All the peaks in the pattern could be indexed to a wurtzite-structure AlN (h-AlN) crystalline structure with lattice constants of *a* = *b* = 0.3111 nm and *c* = 0.4979 nm (JCPDS: 25-1133), which shows that pure wurtzite-AlN can be prepared by arc discharge.

**Figure 2:**
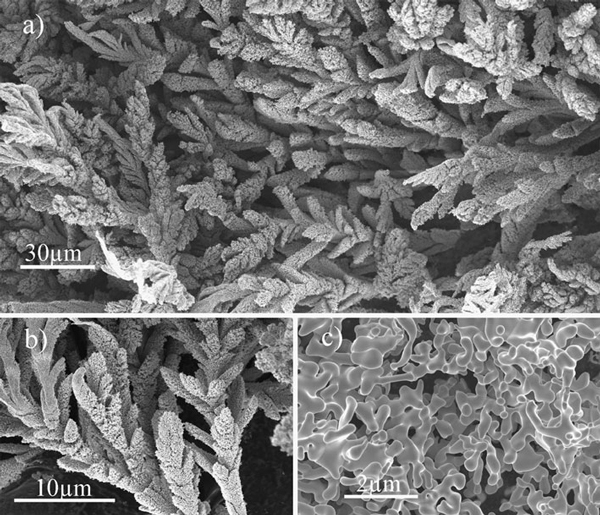
**Typical SEM images of the coral-like AlN nanostructures: a a low-magnification SEM image of high-density as-grown coral-like products; b shows a clear dendritic structure; c a close view of fine branches with melt-out surface**.

A nice coral-like microstructure was prepared using 180 A discharge current for 60 s, as illustrated in Figure 2. The as-grown AlN "coral" has a clear dendritic structure. The length of the trunk is about several tens of micrometers, and diameters are less than 10 μm. Figure 2c shows the fine structure of the coral that has a smooth melt-out surface. The small branches, shown in Figure 2c, have a length of several micrometers, and the diameter is about several hundreds of nanometers. The melt-out surface of the small branches means that the temperature was too high that AlN crystals began to melt.

**Figure 3:**
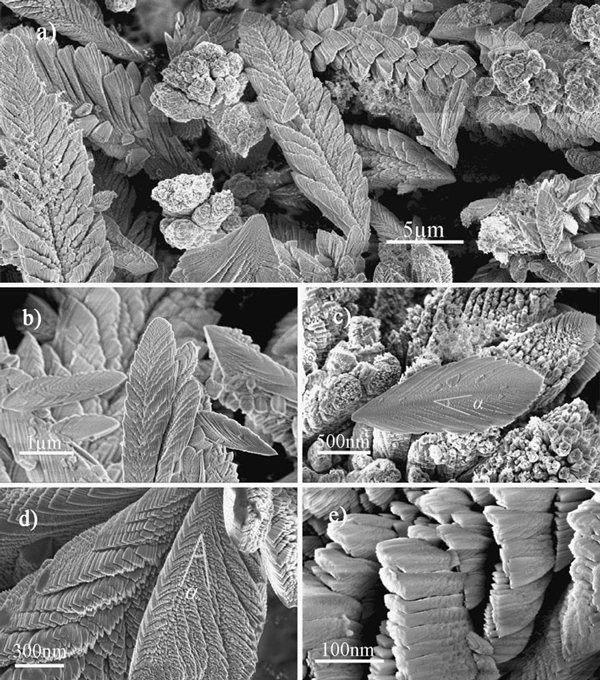
**SEM images of the feather-like AlN nanostructures: a a low-magnification SEM image; b, c typical SEM images of a complete feather-like AlN nanostructure; d, e high-magnification SEM images show a scale-shaped fine structure of the feather-like products**. The angle between the trunk and its branches (*α*) marked in (**c**) and (**d**) is about 30°.

**Figure 4:**
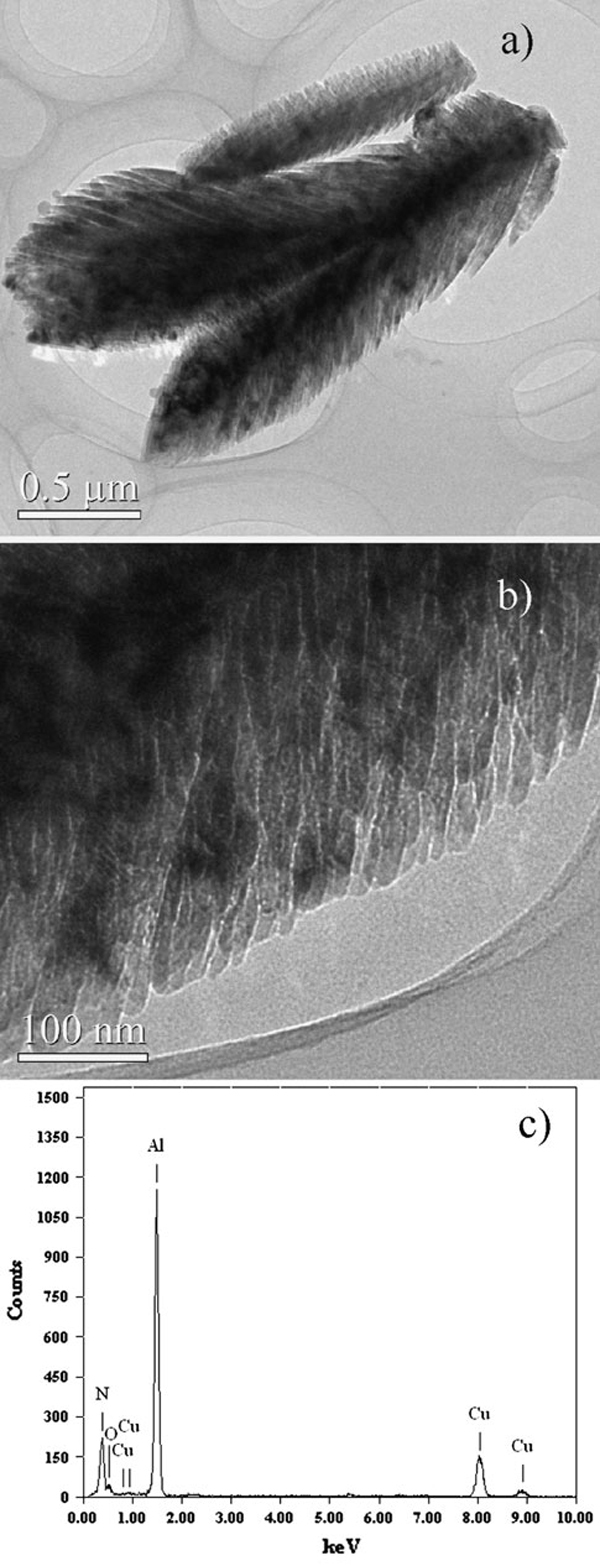
**a, b TEM images of the feather-like nanostructures; c typical EDS pattern of the TEM samples**.

When keeping the current 180 A and decreasing time to 30 s, an amazing self-assembled feather-like nanostructure was prepared as shown in Figure 3. The AlN "feathers" are considered as a hierarchical dendritic nanostructure. Figure 3a is an overall SEM image of feather-like nanostructures with sizes ranging from 2 to 20 μm, which are smaller than the coral-like products. Figure 3b and 3c are typical SEM images of complete feathers in different scales, both of which show a dendritic structure. It implies that the feather-like nanostructure will be transformed to coral-like products by extending the discharge time. Feathers of different sizes observed in the experiments might be at different growth stages. Figure 3d and 3e are high-magnification SEM images, which show more details of feather-like nanostructure. The surface of the feather has a fish scale structure. The scale-shaped fine branches are almost uniform, which are about 100 nm long, 10 nm thick and several tens of nanometers in width. However, there might be more fine structures in the "scale" as shown in Figure 3e. High-resolution TEM was employed to investigate the novel nanostructure, but it was very difficult to get a clear HRTEM image and the corresponding selected area electron diffraction (SAED) pattern of the special nanostructures for the branches cross over each other in 3D space. Figure 4a and 4b are the most clear-cut TEM images we got in the experiments, which supply a better view of the internal details of the feather-like nanostructure. It appears that the feather is constituted of trunk and oblique branches. The trunk is nearly straight, and the angle between the trunk and its branches is about 30°, which is consistent with the SEM results (α marked in Figure 3c, d). The fine branches with a thickness less than 10 nm are separated by void. EDS analyses were carried out on numerous samples and showed that the ratio between Al and N atoms is nearly 1, which ensure that the composition of the feather nanostructure is AlN. Figure 4c is a typical EDS pattern of the TEM samples, and very low levels of oxygen have also been detected in our experiments.

As far as we know, there are very few reports about feather-like nanostructure. Similar "leaf-like" or "dendrite-like" structures have been prepared by MOCVD [19], CVD [20] and magnetron sputtering [21,22] in TiO_2_, W and FeSiAl(Ti/Ta)(O)N thin films. Brien [23] has done a pioneering work on the growth mechanism of the feather-like nanostructure in AlN thin film prepared by reaction sputtering, which confirmed that the trunk was along the [0001] direction and the growth direction of oblique branches was perpendicular to the {10$\bar{1}$3} is 31.65°. Our SEM and TEM images have revealed that the angle between the trunk and its branches is about 30°, and the XRD pattern (Figure 1) shows that both (0001) and (10$\bar{1}$3) have relatively strong peaks. However, feather-like AlN nanostructure prepared by arc discharge is more complex than that had been prepared in the film. The complex 3D feather-like nanostructure has a hierarchical structure, which seems that new small branches with a certain orientation (still about 30°) will grow on the oblique branches.

**Figure 5:**
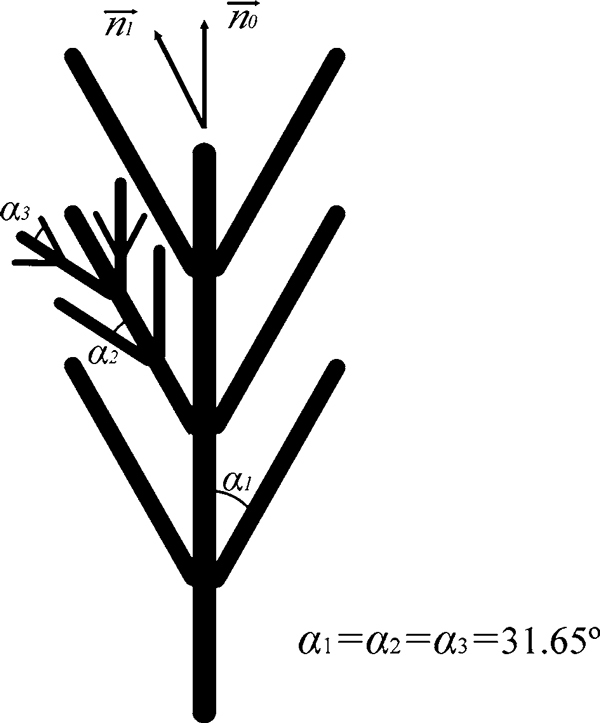
**A scheme of the alternate growth mechanism**. ${\vec{n}}_{1}$ is the direction perpendicular to (10$\bar{1}$3) and ${\vec{n}}_{0}$ is perpendicular to (0001). The theoretical angle (*α*_1,2,3_) between the trunk and its branches is 31.65°.

An alternate growth model has been proposed to explain the novel nanostructure as illustrated in Figure 5. The arc discharge reaction growth should be a VS process. When arc discharge begins, activate Al and N atoms will be continuously generated near the surface of the Al ingot, and a pit will appear immediately as a molten pool. After intense reaction, a lot of AlN crystals will nucleate on the inter surface of the pit. As the (0001) plane of h-AlN is the most close-packed plane,a growth along [0001] direction follows the principle of minimum energy. Nevertheless, the growth is under the control of thermodynamic and kinetic principles simultaneously. During arc discharge, the reaction temperature will be about 2,400–3,000 K and the pressure will become above 1.3 × 10^5^ Pa, which means that the growth process is non-equilibrium and supersaturated. Thus, some oblique growth (perpendicular to the {10$\bar{1}$3} of h-AlN) will happen, which can supply more growth space and high growth speed. Meanwhile, growth along [0001] direction appears on the oblique branches, which means that the nanostructure is formed by an alternate growth along two crystal directions. It should be a balance of the crystal stability and growth speed. The good symmetrical morphology of feather-like nanostructures might originate from the symmetry of the alternate growth model as shown in Figure 5. The outer branch tips had more chance collecting molecules, so more fine structures (like fish scale) were found on the surface, and void was generated by shadowing effect. It should be emphasized that Figure 5 is a simplified 2D diagram, whereas the real branches will grow in a 3D space, which should lead to the complex 3D feather-like nanostructure. Nevertheless, a perfect explanation of the 3D feather-like AlN nanostructures still needs more detailed and direct evidences.

**Figure 6:**
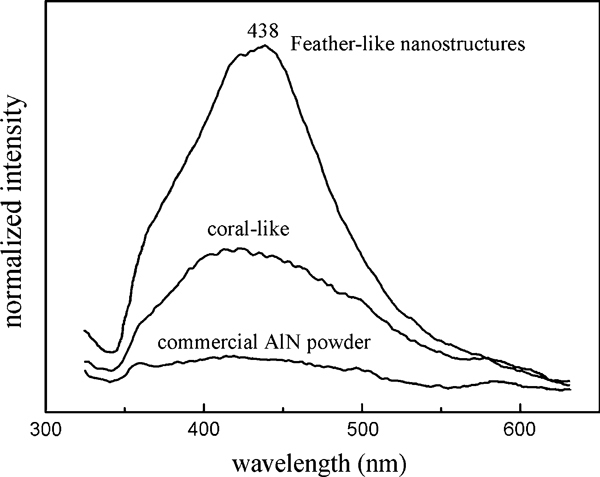
**The photoluminescence spectrums of the feather-like, coral-like products and commercial AlN powders measured at room temperature**. The emission intensities have been normalized by the blue emission peak of the feather-like samples.

Figure 6 shows room temperature photoluminescence of AlN nanostructures prepared in the experiments, and some commercial AlN powders (D50 = 0.5 μm, AlN > 99%, O < 0.4%) were tested for comparison. The emission intensities have been normalized by the blue emission peak of the sample with feather-like nanostructures. The spectrum of the feather-like products shows a strong blue emission band centered at 438 nm (2.84 eV), whereas there is almost no visible light emission generated from the commercial AlN powders. The emission intensity of the coral-like products prepared by longer discharge time has shown a remarkable decrease compared with feather-like nanostructures.

Normally, AlN material has an energy band gap of 6.2 eV, and the band gap should increase when the size of nanostructure decreased [24]. The observed intensive blue emission is obviously not related to the direct band gap transition (larger than 6.2 eV), and it has been attributed to the energy level generated by nitrogen deficiency and oxygen impurities [4,8,12,25] in the wide band gap. Generally, the defects are present at the surface of nanomaterials. The feather-like AlN nanostructures with fine surface structure have a higher surface area to volume ratio than the coral-like products, which results in a high level of surface defects. On the other hand, when increasing the discharge time, more completely reaction and higher growth temperature will make fewer defects left, so a decrease in emission intensity will be observed.

## Conclusion

A complex three-dimensional (3D) feather-like AlN nanostructure was synthesized by a direct reaction of high-purity Al granules with nitrogen using an arc discharge method. By adjusting the arc discharge time, a 3D coral-like AlN nanostructure has also been prepared, which is considered as the evolution of 3D feather-like products. The novel 3D feather-like AlN nanostructure with a hierarchical dendritic structure has shown a good symmetry, and an alternate growth model has been proposed to explain the novel nanostructure. The spectrum of the feather-like products shows a strong blue emission band centered at 438 nm (2.84 eV), which indicates their potential application as blue light-emitting diodes.
